# Large Language Models for Clinical Narrative Processing: Methods, Applications, and Challenges

**DOI:** 10.3390/mps9030069

**Published:** 2026-05-01

**Authors:** Achilleas Livieratos, Junjing Lin, Paraskevi Chasani, Mina Gaga, Fotios S. Fousekis, Charalambos Gogos, Karolina Akinosoglou, Konstantinos H. Katsanos, Margaret Gamalo

**Affiliations:** 1 Independent Researcher, 152 38 Athens, Greece; 2 Takeda Pharmaceuticals U.S.A., Inc., 500 E Kendall St., Cambridge, MA 02142, USA; 3 Department of Neuropathology, Universitätsklinikum Erlangen, Friedrich-Alexander-Universität Erlangen-Nürn berg (FAU), Schwabachanlage 6, 91054 Erlangen, Germany; 4 1st Respiratory Medicine Department, Hygeia Hospital, 151 23 Athens, Greece; 5 Division of Gastroenterology, Department of Internal Medicine, Faculty of Medicine, University of Ioannina School of Health Sciences, 45110 Ioannina, Greece; 6 Department of Medicine, University of Patras, 26504 Rio, Greece; 7 Department of Internal Medicine and Infectious Diseases, University General Hospital of Patras, 26504 Rio, Greece; 8 Pfizer Inc., 500 Arcola Rd., Collegeville, PA 19426, USA

**Keywords:** large language models, clinical practice, electronic health records, medical informatics

## Abstract

Large language models (LLMs) have rapidly advanced natural language processing and are increasingly used to analyze clinical narratives. Their ability to extract information, summarize records, and support clinical workflows makes them potential tools for enhancing documentation efficiency and the secondary application in the analysis of electronic health record (EHR) data. The aim of this work is to synthesize recent evidence on methodological approaches and applications of LLMs for clinical narrative processing, and to assess their performance, benefits, limitations, and implications for clinical practice. Across 2022–2026 studies, LLMs demonstrated strong performance in information extraction, summarization, triage prediction, section classification, and synthetic text generation, often surpassing traditional machine-learning models. Overall, LLMs improved the conversion of unstructured notes into actionable clinical insights, reduced documentation burden, and supported decision-making tasks. Key challenges included hallucinations, variable reproducibility, sensitivity to prompting, domain adaptation gaps, and limited transparency. Our findings indicate that LLMs show substantial promise for transforming clinical narrative processing, but safe adoption requires rigorous evaluation and continuous model auditing. This work provides a structured, non-systematic synthesis of representative studies and is intended as a high-level overview of emerging applications rather than a comprehensive systematic review.

## 1. Introduction

Electronic health records (EHRs) are now common in modern healthcare, containing vast amounts of patient data in both structured and unstructured formats [1,2,3,4]. Although structured fields, such as diagnosis codes and medication lists, are easily captured in some applications, clinicians still depend on free-text clinical notes to document detailed information about patient histories, symptoms, care plans, and details that standard medical coding cannot convey [2,3,4,5,6]. Free-text data cannot be directly processed by traditional computational systems, highlighting the need for natural language processing (NLP) methods to access valuable information hidden in clinical narratives and support clinical reasoning [7,8,9].

Over the years, many NLP techniques have been explored to extract structure from unstructured clinical text. Early methods used statistical text processing, like bag-of-words and topic modeling. Later, they incorporated classical machine learning and sequence models, such as Long Short-Term Memory (LSTM) networks and Convolutional Neural Networks (CNNs), to gather medically relevant information from notes [10,11,12,13]. Recently, transformer-based models like Bidirectional Encoder Representations from Transformers (BERT) have emerged and been fine-tuned on biomedical data, resulting in improved accuracy for clinical text tasks [7,8,9]. However, a persistent issue is that these models often lack generalizability across different clinical settings. As a result, an NLP model trained on notes from one hospital typically does not perform well on notes from another [10,11,12,13]. Previous studies have demonstrated that models trained on one source struggle to recognize sections or concepts in a new context without significant retraining [7,8,9]. Recently, research has shifted to large language models (LLMs) as a possible solution, suggesting that their broad knowledge and contextual understanding might enhance their ability to adapt across domains for tasks such as note section classification [1,14].

Transformers pre-trained on vast general text corpora have delivered state-of-the-art operation on many language understanding tasks. In the biomedical domain, initial transformer models such as BioBERT and ClinicalBERT were trained on domain-specific text, but these had in the order of only 100 million parameters [7,8,9]. By contrast, general-domain LLMs exploded in size—GPT-3 (2020) introduced 175 billion parameters and demonstrated unprecedented capabilities in language generation and few-shot learning [15,16,17,18]. The results showed marked improvements on multiple clinical NLP tasks (e.g., clinical concept extraction, inference, question answering), highlighting that scaling up model parameters and training data can yield significant gains in extracting and understanding clinical narratives [1].

The emergence of general-purpose conversational agents like ChatGPT (GPT-3.5) in late 2022 further catalyzed interest in applying LLMs to clinical practice [19,20,21]. At the same time, the medical community has voiced serious concerns. Notably, LLMs are prone to hallucinations (plausible-sounding but incorrect statements) and may reflect biases present in their training data [15,16]. In a field where accuracy can be life-critical, the tendency of LLMs to sporadically produce fabricated medical content is a significant risk [15,16]. There are also confidentiality and ethical issues: using patient data with LLMs raises privacy questions, and models not specifically designed for healthcare might violate regulatory standards if deployed naively [17]. To address these gaps, researchers have begun developing domain-specific LLMs tailored to healthcare. For example, Peng et al. introduced *GatorTronGPT*, a 20-billion-parameter generative LLM trained on 277 billion words, including 82 billion words of clinical text (drawn from 2 million patients’ EHRs [14]. In one evaluation, physicians could hardly distinguish synthetic notes generated by GatorTronGPT from real human-written notes in terms of readability and clinical relevance [14]. Such findings illustrate both the opportunity and the challenge of LLMs in medicine—they are now capable of producing remarkably human-like clinical text, yet this very ability necessitates rigorous validation to ensure the content is correct and safe.

In summary, early challenges in processing narrative notes are being addressed by transformers and LLMs that bring unprecedented language understanding to the medical domain [22]. Going forward, the most recent developments—exemplified by GPT-4 and other state-of-the-art models—are pushing the envelope in terms of capability, including generating patient-friendly explanations and bridging communication between providers and patients [23]. This work aims to critically evaluate the integration of AI, particularly LLMs, into the analysis and use of clinical notes in healthcare practice. By synthesizing current literature, we aim to identify and synthesize recent applications of LLMs for analyzing clinical narratives as well as to evaluate their performance, strengths, and limitations across key tasks (information extraction, summarization, triage support, section classification, and synthetic text generation). Unlike other work that focuses on specific specialties or isolated applications, we hereby provide a cross-domain synthesis of large language model applications for clinical narrative processing. This structured comparison allows for a broader understanding of how LLM capabilities translate across clinical workflows and highlights areas requiring rigorous validation before implementation. This manuscript is designed as a structured, high-level synthesis aimed at highlighting key trends and challenges in the field, rather than an exhaustive systematic review. This design choice reflects both the rapid evolution of the field and the substantial heterogeneity across studies, which together limit the feasibility and long-term relevance of a fully systematic review approach.

## 2. Materials and Methods

### 2.1. Search Strategy

**Figure 1:**
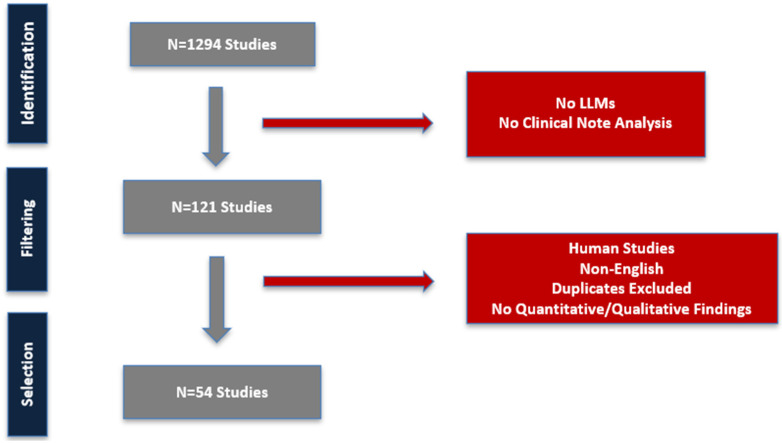
Study Flowchart.

We investigated publications indexed in PubMed from database inception to February 2026. Our search strategy combined clinical terms (e.g., clinical, healthcare, electronic health records, clinical notes) with AI-related terms (e.g., large language models, transformer, GPT, BERT) to identify representative studies. The selection process prioritized conceptual and methodological representation across domains rather than exhaustive coverage, reflecting the exploratory nature of this synthesis. This strategy generated a total of 1294 articles, and 54 studies were ultimately included (Figure 1).

This methodological approach was intentionally designed to reflect the rapidly evolving and heterogeneous nature of the field. Large language model research in clinical applications is characterized by substantial variation in study design, model architecture, datasets, and evaluation metrics, limiting the feasibility of standardized comparison or meta-analysis. Furthermore, the pace of development in this domain means that a fully systematic review risks becoming outdated shortly after completion. Therefore, this work prioritizes conceptual and methodological representation across key application domains rather than exhaustive coverage. The inclusion of both primary studies and evidence syntheses was considered appropriate given the early stage of clinical implementation, where secondary analyses provide important contextual insights into emerging trends, strengths, and limitations.

### 2.2. Eligibility Criteria

Out of the initial corpus retrieved, we filtered studies that demonstrated the use of LLMs or transformer-based models to facilitate clinical note analysis, including summarization, information extraction, section classification, triage/decision support, or synthetic note generation. Inclusion criteria encompassed both primary studies and evidence syntheses evaluating AI or large language model applications for clinical narratives and reporting quantitative or qualitative findings. Eligible studies encompassed human clinical investigations, cross-site evaluations, or in silico clinical note generation and were published in English.

We excluded commentaries and editorials. Animal-only or purely in vitro research without translational relevance was omitted, along with investigations that did not employ AI or ML methods. Articles not available in full text, manuscripts in non-English, duplicates, and conference abstracts were also excluded.

### 2.3. Screening and Data Extraction

Titles and abstracts were screened to assess eligibility, followed by full-text review of selected studies. Three independent reviewers screened articles and resolved disagreements through discussion and consensus. Data were extracted on clinical domain, AI methods employed, input data types (e.g., clinical narratives, structured EHR data, multi-modal inputs), key outcomes, and comparative performance with traditional approaches. Studies were categorized by primary application domain based on the main objective described. Domains included, among others, summarization, information extraction and decision support. Categorization was determined using study objectives, methodology, and reported outcomes. When studies addressed multiple tasks, classification was based on the dominant application reported. Each study was assigned to a single primary domain to ensure consistency.

### 2.4. Model Types

AI models explored in the included studies were transformer-based LLMs (e.g., GPT-4-class systems, ClinicalBERT, GatorTron, CT-BERT). The final synthesis included representative studies meeting all inclusion criteria.

The analysis of the selected studies followed a qualitative synthesis approach. Studies included were systematically categorized based on primary application domain (e.g., summarization, information extraction, decision support). For each study, we extracted information on model architecture, clinical setting, input data type, performance outcomes, advantages, and reported limitations. Comparative evaluation focused on identifying methodological trends, strengths, and weaknesses across domains rather than performing quantitative meta-analysis, due to heterogeneity in tasks, datasets, and evaluation metrics.

## 3. Results

We investigated diverse studies that utilized LLMs for clinical notes across tasks including information extraction, discharge summarization, triage and decision support, section classification, and synthetic text generation.

**Figure 2:**
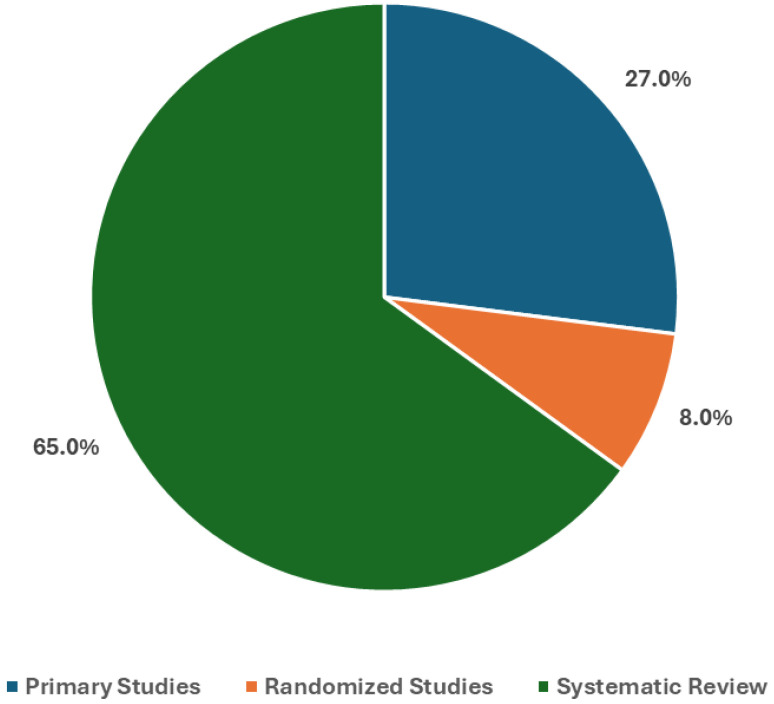
Distribution of study designs to evaluate the utility of LLMs in clinical note analyses.

Figure 2 shows the distribution of study designs that evaluate the use of LLMs in clinical note analyses, as detailed in the literature included [22,23,24,25,26,27,28,29,30,31,32,33,34,35,36,37,38,39,40,41,42,43,44,45,46,47,48,49,50,51,52,53,54,55,56,57,58,59,60,61,62,63,64,65,66,67,68,69,70,71,72,73,74,75]. Most of the sources were systematic, scoping, umbrella, or integrative reviews, making up 65% of the total. This reflects the quick growth of secondary studies assessing the performance, safety, and clinical use of large language models across various healthcare areas [31,32,33,34,35,36,37,38,39,40,41,42,43,44,45,46,47,48,49,50,51,52,53,54,55,56,57,58,59,60,63,64,72]. Primary studies, which include technical model development and observational evaluations, represented 27% of the evidence [22,23,24,25,29,61,62,65,66,67,68,69,70,71,73]. Randomized or prospective interventional trials made up 8%, indicating the limited amount of high-level interventional evidence in the current literature [26,27,28,30].

The high proportion of review articles likely reflects the rapid expansion of interest in LLMs and the early stage of clinical implementation, where synthesis of emerging evidence precedes large-scale primary investigations. Reference lists of selected reviews were not further examined to identify additional primary studies, as those were already captured by the search strategy. Secondary studies were retained, as they provided contextual insights into reported strengths, limitations, and implementation considerations for LLM-based clinical narrative processing.

**Figure 3:**
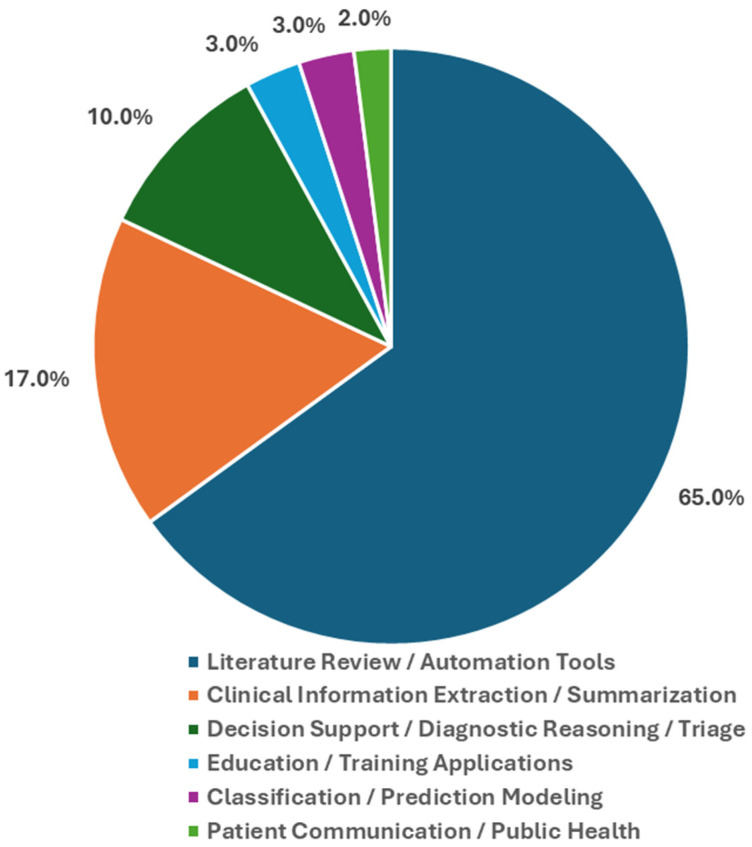
Proportion of studies categorized by LLM use case across health-related domains.

Figure 3 shows the distribution of LLM use cases across the studies included. The largest group consists of literature reviews and general evaluations of LLM applications, making up 65%. This indicates a strong focus on gathering new evidence and surveying LLM capabilities in various clinical areas [31,32,33,34,35,36,37,38,39,40,41,42,43,44,45,46,47,48,49,50,51,52,53,54,55,56,57,58,59,60,63,64,72]. A significant number of studies concentrated on clinical information extraction and summarization, accounting for 17%. This points to a continued interest in automating the interpretation of unstructured clinical text and documentation workflows [22,23,24,25,29,65,66,67,71].

Studies on decision support, diagnostic reasoning, and clinical task assistance made up 10% of the literature. They explored how LLMs could improve clinician performance and frontline care processes [26,28,30,73]. Smaller percentages looked at education and training applications (3%), classification and prediction modeling (3%), and patient communication tools (2%) [61,62,68,69,70,75]. These findings show a wide and quickly developing research area. It combines large-scale evidence synthesis with practical work on clinical implementation and workflow integration.

Table 1 displays representative studies for different tasks. Most of these studies used transformer-based architectures like GPT-2, GPT-4-class models, ClinicalBERT, GatorTron, GatorTronGPT, ChatGLM, and Alpaca. Some studies used ensemble strategies that combined zero-shot LLMs with BERT. Others examined domain-scaled foundation models trained on billions of EHR tokens.

## 4. Discussion

### 4.1. Strengths and Opportunities

The breadth of current research highlights numerous strengths of LLMs in clinical documentation. First, LLM-based systems have demonstrated remarkable capability in summarizing complex narratives. This points to a future in which clinicians spend less time on clerical writing and more on patient care. Second, LLMs offer new opportunities in triage and prognostication. Similarly, researchers have begun using LLMs to parse provider notes and feed the results into prediction models for outcomes or resource needs, indicating that unstructured narrative data can add prognostic value when intelligently harnessed.

Another promising area is the development of patient-facing tools. LLMs like GPT-3.5/4 have shown they can reformulate clinical information in patient-friendly ways [70]. Moreover, LLMs’ multilingual abilities open doors to serving diverse populations: a recent multi-center evaluation found GPT-4 could accurately analyze notes not just in English but also in Spanish and Italian, with physicians agreeing with ~79% of its outputs (and even higher agreement for Spanish) [71]. As models continue to improve, we see expanding opportunities to integrate them into workflows for documentation drafting, decision support, patient communication, and education.

### 4.2. Weaknesses and Risks

A key observation emerging from this synthesis is the uneven maturity of evidence across application domains. Tasks such as summarization and information extraction show relatively consistent performance improvements across multiple settings, whereas decision support and diagnostic reasoning remain supported by smaller datasets and limited prospective validation. This imbalance suggests that LLM deployment in clinical workflows should prioritize lower-risk augmentation tasks before high-stakes decision support applications. Even further, a central concern is the validity gap, a disconnect between controlled evaluation settings and real-world clinical environments. Additionally, the predominance of secondary literature reflects a field in rapid conceptual expansion but still lacking robust interventional trials, underscoring the need for prospective clinical validation studies.

Counterbalancing the optimism, LLMs are prone to hallucinations, which is unacceptable in medical documentation. Even when overall quality approaches that of human clinicians, as with ambient note-generation systems, the AI-produced notes tend to include more inaccuracies: one multi-specialty study found hallucinated content in 31% of AI-generated notes versus 20% of physician-written notes [67]. In Farrag et al.’s work on LLMs for social drivers of health, only 21% of studies validated their model on an external dataset [72]. This illustrates that while LLMs can do astonishing things with text, significant work remains to ensure they do the right things reliably in the clinical context.

### 4.3. Practical Applications

A range of practical applications for LLMs in clinical note analysis and workflow are rapidly emerging. Given the extensive length of many notes, summarizing key information can greatly aid both clinicians and patients in understanding a case. Recent studies have leveraged LLMs like GPT-4 to generate coherent and contextually relevant summaries of patient encounters [22,23]. Beyond summarization, LLMs are being applied to information extraction tasks on clinical notes. In a specialized domain example, Chiang et al. showed that a generative LLM (GPT-2 based) fine-tuned on neurology notes was able to accurately extract the frequency of patient headaches (a key outcome metric in migraine management) from text, far outperforming a conventional ClinicalBERT model on the same task [23]. LLMs have also been explored for clinical reasoning and decision support using notes as input. Experiments previously employed ChatGPT to read, for example, emergency department (ED) physicians’ notes and generate a differential diagnosis list as a means of assisting diagnostic reasoning [73,74,75,76,77]. These examples underscore the versatility of artificial intelligence systems in handling diverse clinical text tasks—from compressing long narratives into summaries, to extracting structured facts, to performing higher-level reasoning and prognostication based on unstructured inputs [78,79,80].

### 4.4. Challenges and Safeguards

Equally important to the successes of LLMs in healthcare are the challenges and safeguards needed for their responsible integration into clinical practice [15,16,78]. One concern is that the performance of LLMs can vary across different clinical scenarios; an LLM that excels at one task may falter on another without careful tuning [15,16,78]. This variability necessitates extensive validation and often task-specific adaptation to ensure reliability in each intended use. A related issue is that clinical notes contain domain-specific jargon, abbreviations, and context that models must learn [78,81]. Large models partially address this by ingesting vast amounts of clinical text, but ongoing domain adaptation (and possibly expert curation of training data) is required to handle evolving medical language and rare edge cases [81]. Furthermore, the accuracy and safety of LLM outputs remain a pressing concern. Studies have found that LLM-generated clinical texts can suffer from omissions; for example, nearly 40% of AI-crafted discharge summaries in one evaluation had potential safety issues due to missing content [15,16]. There is also movement toward establishing regulatory oversight for AI clinical tools, meaning LLM systems may eventually require certification or benchmarking against clinical safety criteria before routine use [25,82].

Beyond technical performance and workflow integration, the adoption of LLMs in clinical practice raises critical challenges. One important but underexplored issue is digital illiteracy across generations of clinicians [31,40]. Without targeted education and training, this generational divide risks creating uneven uptake, inappropriate reliance, or underutilization of LLM-based systems [31,40]. Unequal access across healthcare settings further compounds these challenges [31,40]. The long-term effects of cognitive offloading whereby clinicians increasingly rely on such systems for reasoning and documentation remains unclear [31,40]. When combined with LLM limitations such as hallucinations, insufficient understanding may act as a risk multiplier, increasing the likelihood of inappropriate reliance on model outputs.

Bias remains a persistent and structurally embedded risk [16]. Many LLMs are trained on datasets that underrepresent minority populations, certain disease phenotypes, or non-Western healthcare contexts [16]. Limited training input from underrepresented populations can lead to systematic bias, misinterpretation of symptoms, or inappropriate recommendations, thereby reinforcing inequities in care delivery [16]. Moreover, the rapid evolution of medical knowledge introduces additional complexity [40]. Clinical guidelines, diagnostic criteria, and therapeutic standards change frequently, yet many LLMs are static once deployed [40]. This raises important questions about how quickly models can be updated, how knowledge cut-offs are managed, and how outdated or superseded recommendations are prevented from influencing clinical decisions. Furthermore, the growing use of LLM-generated data introduces the risk of model collapse or degenerative feedback loops, where future models are trained on outputs produced by earlier models, potentially amplifying errors and reducing information quality [83]. Finally, legal and accountability issues remain unresolved. In cases where an LLM contributes to a clinical error, responsibility is unclear: liability may fall on the clinician, the institution, the software developer, or a combination thereof [48]. This ambiguity poses substantial medico-legal risk and may hinder adoption.

### 4.5. Limitations

This work has several limitations that must be acknowledged. First, our scope was confined to English-language publications up to February 2026, which may have excluded relevant non-English studies or very recent findings that emerged thereafter. The fast-moving nature of the LLM field means new results (and even new models) are appearing continually; our snapshot of the literature may quickly become dated as the technology evolves. Second, we focused on studies applying LLMs specifically to clinical note practice and reporting quantitative evaluations. We excluded commentary and purely theoretical papers, which helped concentrate on empirical results but risks overlooking important perspectives or ethical analyses in the broader discourse. There is also the possibility of publication bias—successful applications of LLMs are more likely to be published than negative or neutral studies, skewing the overall impression of progress. We attempted to mitigate this by including a range of use cases and noting challenges reported, but we cannot be certain we captured unpublished difficulties teams may have encountered. Applying LLM-assisted analysis to the studies included could provide complementary insights, including automated categorization of tasks, extraction of performance metrics, and identification of methodological patterns. However, such approaches require careful validation, as LLM-based synthesis may introduce bias, hallucinations, or inconsistencies if not properly controlled. Third, the heterogeneity of included studies limited our ability to directly compare performance. Different works evaluated LLMs on varied tasks, with disparate metrics and gold standards, so our synthesis remained qualitative. Finally, while we aimed for a comprehensive search, it is possible we missed some relevant studies in subspecialty domains or industry settings that are not indexed in the databases used. This work is not intended to represent a systematic review and does not aim to comprehensively capture all available literature. The chosen approach reflects a deliberate focus on conceptual synthesis rather than exhaustive coverage, which is appropriate for a rapidly evolving and heterogeneous research area.

## 5. Conclusions

LLMs are rapidly reshaping how clinical narratives are processed, interpreted, and used within healthcare systems. This work highlights that LLMs already demonstrate strong performance in tasks such as information extraction, summarization, clinical coding, and error detection, with accuracy often comparable to or exceeding traditional NLP approaches. Their capacity to interpret unstructured text at scale offers clear opportunities to reduce workload, improve data quality, and support clinical decision-making. However, these gains coexist with persistent challenges, including hallucinations, variable domain adaptation, opacity of decision processes, and concerns regarding privacy, security, and regulatory compliance. Current evidence also shows that model performance is highly sensitive to prompting strategy, training data, and clinical context.

As LLM integration in healthcare accelerates, responsible deployment will require rigorous evaluation frameworks, transparent reporting standards, and continuous model auditing. Ultimately, LLMs should be viewed not as replacements for clinical expertise but as augmentative tools that can enhance efficiency and support high-quality patient care. Continued research, thoughtful governance, and real-world validation will determine the extent to which LLMs fulfil their promise in transforming clinical narratives into actionable clinical intelligence.

## Figures and Tables

**Table 1:** Summary of representative primary research studies evaluating the use of LLMs across different clinical narrative processing tasks.

Study (Authors, Year)/Task	Technology Used (Clinical Notes Task)	Setting	Advantageous Output	Limitations
Chiang et al., 2024 [23] Information extraction	GPT-2–based generative NLP model fine-tuned on headache specialist clinic notes. Compared against ClinicalBERT for extracting headache frequency from notes.	Extracted migraine headache frequency from unstructured neurology notes.	92% accuracy, outperforming a BERT-based model. Enabled automated conversion of narrative text into structured clinical data, reducing manual chart abstraction and improving the reliability of headache outcome tracking.	Trained and evaluated only on notes from one specialty (Mayo Clinic headache clinics), so generalizability to other settings may require re-training or fine-tuning. Complex implicit statements (requiring arithmetic reasoning) still posed challenges—the model could miscalculate frequency if not explicitly stated.
Gao et al., 2025 [65] Decision support/Triage prediction	Multi-LLM pipeline (ChatGLM-2, GLM-4, Alpaca-2) with prompt templates and retrieval augmentation, used to parse emergency department triage notes (chief complaint and anamnesis). Output a severity score, which is fed into a logistic regression predictor.	LLM-derived severity scores from chief complaint and anamnesis text.	Significantly improved early identification of critically ill ED patients. Unstructured note content can yield actionable early warning signals that enhance triage decision-making and complement structured EHR data.	Diminishing returns at later stages of care: once full information (e.g., vital signs) was available, the added benefit of the LLM-based score was modest and, in some scenarios, not statistically significant. The LLMs were not fine-tuned on medical data (due to limited data and computational resources), relying on prompt-based use of pre-trained models.
Oliveira et al., 2025 [22] Summarization/Documentation assistance	GPT-4-powered discharge summary generation system, with prompts and iterative feedback from physicians and patients. The LLM produces draft hospital discharge summaries from inpatient notes, which were evaluated against human-written summaries.	GPT-4-generated discharge summaries demonstrated near-human accuracy in capturing diagnoses and hospital course.	By producing coherent, clinically relevant draft summaries, the system reduces clinician documentation burden and provides a reliable starting point for finalizing discharge documentation.	Preliminary qualitative evaluation—the assessments relied on user feedback and were not blinded or independently validated, introducing potential bias. The study used a limited dataset, and the system’s performance in a large-scale or real-time clinical workflow remains untested.
Yang et al., 2022 [1] Fine-tuning foundational language modeling for clinical NLP tasks	*GatorTron*: a large clinical language model (8.9 B parameters) trained from scratch on >90 B words of EHR text (de-identified) using a transformer architecture. Evaluated on core clinical NLP tasks (extraction, textual similarity) to test benefits of scale.	Achieved substantial accuracy gains (≈9–10% absolute improvement) across core clinical NLP tasks such as concept extraction, inference, and medical QA.	These improvements support more reliable automated interpretation of clinical narratives and enable downstream applications that transform raw EHR text into actionable insights.	The largest model (8.9 B) was still an order of magnitude smaller than cutting-edge general LLMs (e.g., GPT-3 has 175 B parameters), so there may be further unrealized gains from scaling up model size. The study focused on benchmark tasks with structured outputs; it did not explore generative applications like free-text summary or dialogue.
Zhou et al., 2023 [24] Section classification	Clinical note section classification using an *LLM* (*FLAT-T5*, *BioMedLM*, *Galactica*) *+ BERT ensemble*. An open-source LLM (zero-shot) was prompted to assign section labels (SOAP categories) to each section of a note, and its outputs were combined with a domain-specific BERT classifier’s predictions. Tested on notes from multiple hospitals to evaluate cross-site transfer.	Zero-shot LLM predictions combined with a BERT classifier improved cross-hospital section classification accuracy, especially in out-of-domain settings.	Facilitates automated structuring of heterogeneous clinical notes, reducing preprocessing effort and improving the usability of narrative text for downstream analytics.	Due to data-sharing constraints, the study used only open-source LLMs and could not evaluate proprietary models like GPT-4 on the task. The set of LLMs tested was not exhaustive—new models are continually emerging, and results might vary with a more powerful LLM or different prompt designs.
Wang et al., 2024 [68] Diagnosis-Related Group prediction	DRG-LLaMA to predict hospital Diagnosis-Related Groups (DRGs). Compared against ClinicalBERT and CAML baselines for both single-label and two-label classification tasks.	Improved prediction of Diagnosis-Related Groups from clinical notes compared with ClinicalBERT and CAML baselines.	Enhanced DRG classification supports more accurate billing, reduces administrative burden, and demonstrates how narrative text can directly inform operational decision-making.	Computationally restricted to ≤13 B parameters, with no hyperparameter search. Imbalanced DRG distribution and lack of external validation limit generalizability. Errors arose from incomplete information and inadequate clinical concept extraction.
Hussain et al., 2025 [69] Multidimensional sleep health extraction from clinical notes	Systematic evaluation of prompted and fine-tuned LLMs and a fine-tuned encoder-based classifier (ModernBERT) for detecting nine sleep health categories (e.g., timing, duration, efficiency, disorders, sleepiness, interventions, medication, behavior, satisfaction).	A fine-tuned ModernBERT classifier outperformed both prompted and fine-tuned LLMs for detecting nine sleep health categories in clinical notes, achieving the highest overall performance.	This demonstrates that an efficient encoder-based model can reliably convert full-length sleep-related notes into structured labels suitable for large-scale surveillance and research.	Models showed bias across gender and race. Performance varies by department and class, with sleep efficiency and daytime sleepiness hardest to classify. Small dataset size and domain imbalance limited generalizability. Fine-tuned LLMs exhibited hallucinations and latency issues, and generative models were inefficient for structured clinical deployment.

## Data Availability

No new data were created or analyzed in this study. Data sharing is not applicable to this article.
